# Evaluating the energetics of entrainment in a human–machine coupled oscillator system

**DOI:** 10.1038/s41598-021-95047-x

**Published:** 2021-08-04

**Authors:** Ryan T. Schroeder, James L. Croft, John E. A. Bertram

**Affiliations:** 1 Biomedical Engineering, University of Calgary, Calgary, AB Canada; 2 School of Medical and Health Sciences, Edith Cowan University, Perth, WA Australia; 3 Faculty of Kinesiology, University of Calgary, Calgary, AB Canada; 4 McCaig Institute for Bone and Joint Health, Cumming School of Medicine, University of Calgary, Calgary, AB Canada

**Keywords:** Biophysics, Biomedical engineering, Oscillators, Bioenergetics

## Abstract

During locomotion, humans sometimes entrain (i.e. synchronize) their steps to external oscillations: e.g. swaying bridges, tandem walking, bouncy harnesses, vibrating treadmills, exoskeletons. Previous studies have discussed the role of nonlinear oscillators (e.g. central pattern generators) in facilitating entrainment. However, the energetics of such interactions are unknown. Given substantial evidence that humans prioritize economy during locomotion, we tested whether reduced metabolic expenditure is associated with human entrainment to vertical force oscillations, where frequency and amplitude were prescribed via a custom mechatronics system during walking. Although metabolic cost was not significantly reduced during entrainment, individuals expended less energy when the oscillation forces did net positive work on the body and roughly selected phase relationships that maximize positive work. It is possible that individuals use mechanical cues to infer energy cost and inform effective gait strategies. If so, an accurate prediction may rely on the relative stability of interactions with the environment. Our results suggest that entrainment occurs over a wide range of oscillation parameters, though not as a direct priority for minimizing metabolic cost. Instead, entrainment may act to stabilize interactions with the environment, thus increasing predictability for the effective implementation of internal models that guide energy minimization.

## Introduction

Human walking is an oscillating system where the body moves in cyclic patterns to traverse a substrate—often a static environment, e.g. a sidewalk. However, sometimes the environment behaves as a second oscillating system. In this case, the human and the environment together constitute a coupled oscillator system. For example, pedestrians sometimes spontaneously synchronize the frequency of their steps with that of a swaying bridge as they cross^1^, and this frequency matching is referred to as entrainment. Laboratory experiments have demonstrated similar gait entrainment when individuals are asked to walk on treadmills actuated with controlled oscillations in mediolateral^2,3^ and vertical directions^4,5^. However, some of these studies have shown entrainment to be an uncommon response^3^. In a different experimental paradigm, human entrainment was demonstrated with periodic electrical stimulations of the medial gastrocnemius while walking on a conventional treadmill^6^. Furthermore, infants have shown the ability to learn entrainment of their bouncing frequency with the resonance of an elastic harness^7^. Humans are also sensitive to spontaneous entrainment with auditory stimuli—e.g., when adapting running cadences to adjusted music tempos^8,9^.

Ahn and Hogan^10,11^ interpreted subject entrainment with an ankle exoskeleton as evidence that human locomotion is controlled, at least in part, by low level rhythmic nonlinear oscillators (e.g. central pattern generators). In their study, an actuator provided periodic torque profiles at the ankle joint independent of the subject’s actions. Over time, subjects learned to entrain with the periodicity of the exoskeleton and align muscle activation with that of the artificial system. It is unclear whether such behaviors represent a simple quirk of control mechanisms driving rhythmic gait, or if entrainment patterns are evidence of a more active process guiding effective locomotion in dynamic environments.

A longstanding perspective on gait recognizes that preferred movement patterns are largely consistent with energy minimization over a wide range of circumstances. There is strong empirical evidence that individuals naturally select walking parameters (step frequency, step length, step width, etc.) that minimize metabolic energy per distance travelled^12–15^. Furthermore, it appears that internal models are continuously updated in real time to optimize energy output under novel circumstances^16–18^.

**Figure 1:**
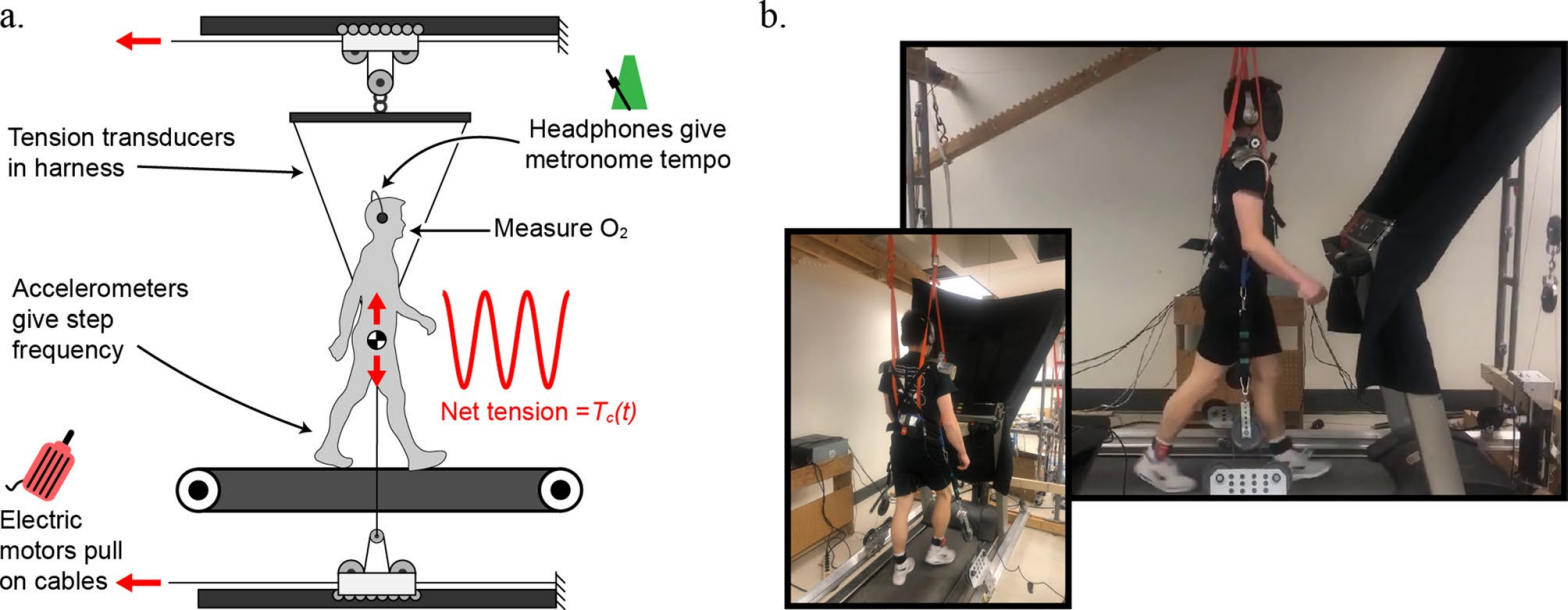
System schematic and images. (**a**) A schematic of the oscillator system is depicted in the sagittal plane. (**b**) Images of a subject walking in the system during a trial, from the side and from behind. Downward force came as the resultant of self-equalizing oblique cables. A curtain was used to blind the subject from any motion of the pulleys or motors, and headphones were used to play ambient noise to block out rhythmic sounds from the system. The headphones were also used to play a metronome beep during portions of the experiment. A more detailed description of the system can be found in the Supplementary Materials.

Here, we test if entrainment to external oscillations is motivated by a reduction in metabolic expenditure. To accomplish this, we used a mechatronics oscillator system to provide periodic vertical forces (upward and downward) to the trunks of subjects as they walked on a treadmill (Fig. 1). As opposed to an exoskeleton strapped to a single joint, the oscillation system in this study is used to more directly influence the center of mass (CoM) and its dynamics—arguably a fundamental aspect of the task of locomotion^19^—to directly assess locomotor control strategies driving interactions within a dynamic environment.

The system used two linear servomotors to tug on a pulley-cable system connected to a body harness worn by subjects (Fig. 1). An open loop current control was prescribed to the motors during experiments where the oscillation frequency and amplitude were fixed at different values for each trial condition. A more detailed description of the system can be found in the Supplementary Materials.

**Figure 2:**
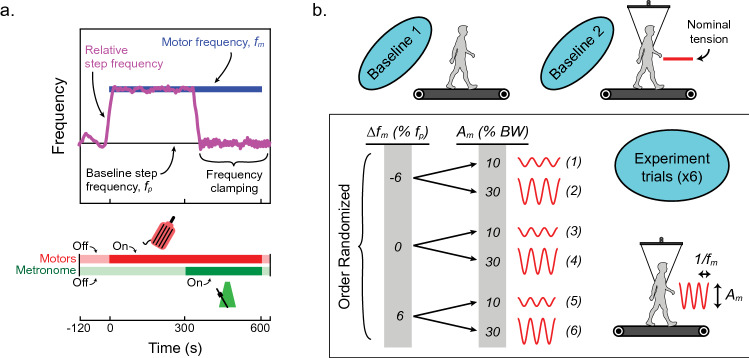
Experimental protocol. (**a**) A generic trial condition is depicted with example step frequency data over time (magenta) and constant motor frequency (blue). Subjects walk with no oscillations during the first 2 min of the test. Motor oscillations begin at time zero and continue for 5 min while subjects freely interact with the system. Oscillations continue for another 5 min, but now a metronome directs individuals to step at their baseline preferred frequency ($f_{p}$) despite the external oscillation frequency (“frequency clamping”). The oscillations and metronome are terminated, and the subject is given fifteen additional seconds to prepare for the end of the trial. (**b**) Baseline conditions and oscillation parameters prescribed to the system during experiment trials are shown: ${\Delta }f_{m}$ is the percent difference in oscillation frequency relative to subjects’ baseline preferred step frequency (${\Delta }f_{m} = 0, \pm 6\%$) and $A_{m}$ is the oscillation amplitude expressed in units of subject body weight (BW) ($A_{m} = 10, 30\%$). Trial conditions were implemented randomly to reduce ordering effects.

The experiment was divided into two phases while external oscillations were present (Fig. 2): first, subjects walked with a freely-chosen step frequency in response to the oscillations (i.e. individuals were allowed to entrain to the oscillations); second, individuals were instructed to follow the beat of a metronome programmed to subject-specific baseline frequencies, measured a priori. Both experiment phases lasted 5 min and allowed for a comparison of energetic consumption during entrainment and non-entrainment. Although previous research has shown small reductions of oxygen consumption in the presence of a regulating auditory cue such as a metronome^20^, we assumed the energetic effect of mechanical entrainment with large force oscillations to be much larger than any potential effects of metronome entrainment. In order to characterize subject-preferred mechanical interactions with the oscillation system, phase alignment of force oscillations relative to the step cycle were measured when the metronome was inactive.

## Results

### Subjects entrain to external oscillations

**Figure 3:**
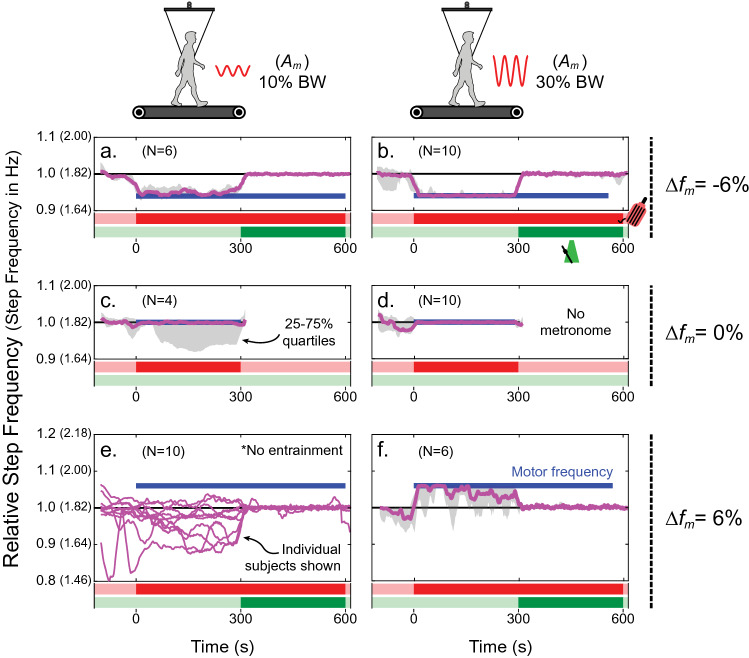
Entrainment results. The median relative step frequency ($f_{r}$, step frequency divided by preferred step frequency in Baseline 2; magenta) of all subjects who entrained (N) is plotted over each trial duration. Step frequency (in Hz) is shown for the average subject in parentheses. Twenty-five and 75% quartiles are used to indicate the distribution at every time point (grey shaded area). All trial conditions are shown, including: ${\Delta }f_{m} = - 6\%$ in (**a**) and (**b**); ${\Delta }f_{m} = 0\%$ in (**c**) and (**d**); ${\Delta }f_{m} = 6\%$ in (**e**) and (**f**); $A_{m} = 10\% \;{\text{BW}}$ in (**a**), (**c**) and (**e**); $A_{m} = 30\% \;{\text{BW}}$ in (**b**), (**d**) and (**f**). The oscillations began at ${\text{Time}} = 0\;{\text{s}}$ and ended at approximately ${\text{Time}} = 600\;{\text{s}}$. During $0 \le {\text{Time}} \le 300\; {\text{s}}$, subjects responded freely to the force oscillations. During $300 \le {\text{Time}} \le 600\;{\text{s}}$, subjects were directed to follow the cadence of the metronome at their predetermined baseline step frequency (“frequency clamping”) even as the oscillations continued at a different frequency. There was no metronome used in trials where ${\Delta }f_{m} = 0\%$, since frequencies were already matched. As a result, these experiments ended after around ${\text{Time}} = 300\;{\text{s}}$. Note, median data are only shown for individuals who entrained at least once throughout the trial. In the trial condition where ${\Delta }f_{m} = 6\%$ and $A_{m} = 10\% \;{\text{BW}}$, individual subject data are shown instead since no entrainment occurred.

Figure 3 shows the median step frequency (magenta) as well as 25% and 75% quartiles (grey shaded region) for individuals who entrained their steps with external oscillations at least once during the indicated trial condition. Subjects exhibited entrainment in 36 out of 60 total trials (60%, where the total number of trials is given by six conditions times ten subjects). However, the level of entrainment varied between individuals; e.g. some only entrained in two trials while others entrained in five out of six total trials. The likelihood of subject entrainment largely depended on the oscillation parameters prescribed: frequency, $\Delta f_{m}$, expressed as a percent difference from subject baseline and amplitude; $A_{m}$, expressed as a percentage of subject body weight (BW) force (see Methods for details). For example, all ten subjects entrained when ${\Delta }f_{m} = - 6, 0\%$ and $A_{m} = 30\% \;{\text{BW}}$ (Fig. 3b,d). Conversely, no subjects entrained when $\Delta f_{m} = 6\%$ and $A_{m} = 10\%\; {\text{BW}}$, thus only individual subject data are shown (Fig. 3e). In general, entrainment in conditions with higher motor frequencies and lower amplitudes was less stable and more transient. Note, the data for $\Delta f_{m} = 0\%$ end earlier than other trials since there was no metronome used (the oscillation frequency already matched baseline preferred, so was deemed unnecessary; Fig. 3c,d). However, individuals largely followed the metronome in other trials, as the median data quickly converged on a relative frequency of one at approximately 300 s into the trial. While the metronome was active, the root mean square error (RMSE) comparing subject step frequency to the metronome frequency ranged between 0.034–0.059 Hz, or 1.8–3.3% of the metronome frequency, depending on the trial condition.

**Figure 4:**
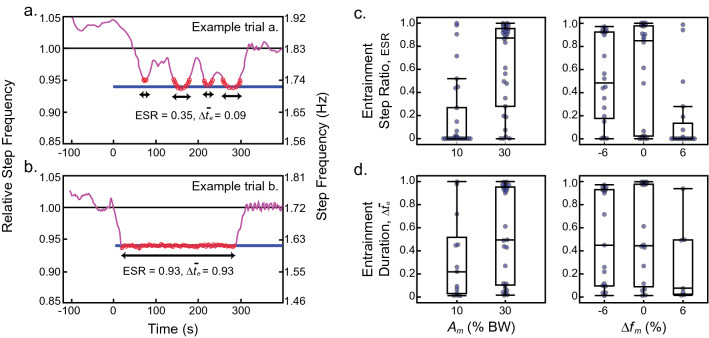
Entrainment is sometimes transient. (**a**) Data from an example trial illustrates transient entrainment where step frequency (magenta) oscillates towards and away from the motor frequency (blue). Red data points indicate when the subject is considered entrained with the oscillator system (see “Methods” section for details on entrainment definition). (**b**) Data from an example trial illustrates steady entrainment. (**c**) The entrainment step ratio (ESR; ratio of entrained steps to total steps taken during the first 5 min of oscillations in the experiment) and (**d**) the average entrainment duration (${\Delta }\overline{t}_{e}$; average time duration of bouts of entrainment) are shown as a function of oscillation amplitude and motor frequency, where each data point represents a subject’s level of entrainment during each trial and box plots summarize the distribution. Linear mixed models were used to statistically test the effects of trial conditions on both entrainment metrics shown here (see Table S1 in the Supplementary Materials for full results).

In many instances, subjects exhibited consistent entrainment (Fig. 4b). However, sometimes a more transient entrainment response was observed—meaning their step frequency drifted in and out of the oscillation frequency throughout the trial (Fig. 4a). To better characterize how well subjects entrained their gait in the various trial conditions, two metrics were considered: entrainment step ratio (ESR, Fig. 4c) and average entrainment duration ($\Delta \overline{t}_{e}$, Fig. 4d). The entrainment ratio is the proportion of steps within ± 3 standard deviations (SD) of the motor frequency (1 SD determined from step frequency data in Baseline 2) during the first 5 min of exposure to oscillations in the experiment (i.e. no metronome). However, because this metric does not consider how bouts of transient entrainment are distributed throughout the trial, $\Delta \overline{t}_{e}$ indicates the average time duration of all bouts in a given trial as a fraction of the 300 s time allotted. Thus, for both entrainment metrics, a value of zero means that no entrainment occurred while a value of one means that subjects entrained throughout the entire trial.

Linear mixed models indicated increases in the level of entrainment between 10 and 30% BW oscillation amplitudes: 35–56% more steps entrained during experiments (effect of $A_{m}$ on ESR, $p < 0.001^{*}$) and the average duration of entrainment bouts increased by 0.20–0.49 or 60–148 s (effect of $A_{m}$ on $\Delta \overline{t}_{e} , \;p < 0.001^{*}$), depending on the oscillation frequency. Conversely, higher oscillation frequencies led to decreases in the level of entrainment when ranging from − 6 to + 6% baseline preferred frequency: 13–23% less steps entrained during experiments (effect of $\Delta f_{m}$ on ESR, $p < 0.001^{*}$) and the average duration of entrainment bouts decreased by 0.09–0.24 or 27–71 s (effect of $\Delta f_{m}$ on $\Delta \overline{t}_{e} ,\; p = 0.001^{*}$), depending on the oscillation amplitude. An interaction between oscillation frequency and amplitude was not significant ($p = 0.273$). In fact, this interaction was not significant in any of the models tested. Figure 4c,d illustrate higher levels of entrainment at larger amplitudes and lower motor frequencies, despite large inter-subject variation overall.

### Entrainment does not reduce metabolic power

**Figure 5:**
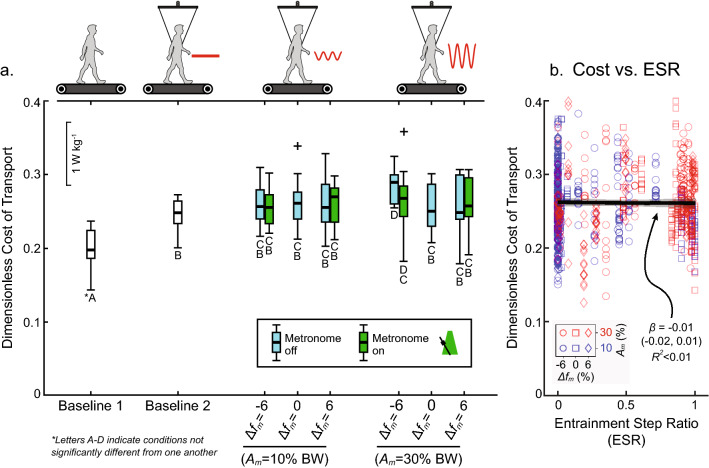
Metabolic power does not depend on entrainment. (**a**) Non-dimensional metabolic power was compared by oscillation parameters and metronome status (on or off) using a mixed linear regression. A post hoc Tukey Honestly Significant Difference test was used to compare estimates of metabolic power in the model. Box plots are labelled with letters (e.g. A–D) indicating conditions where power is not significantly different. Outliers are marked with “+”. See Table S2 in the Supplementary Materials for full results. Baseline 1 and 2 refer to walking on the treadmill without and with the harness, respectively. Box plots are shown for all metabolic data collected during the first 5 min of oscillations where the subject freely responded to the oscillation forces (i.e. metronome off). During the next 5 min of oscillations, a metronome guided subjects to step at their baseline preferred frequency (importantly, not matched to the oscillation frequency). This allowed for metabolic cost comparisons between entrained and non-entrained gait. (**b**) Entrainment step ratio (ESR) is directly assessed for its effect on metabolic power during experimental phases where the metronome was inactive. All data points are shown for all subjects in all trials (754 measurements in total). The slope ($\beta$) is not significantly different from zero ($p = 0.393$; see Table S1, Model 3. for full results) indicating no effect of entrainment level on energetic cost.

Metabolic power was compared for individuals in all trial conditions with the metronome turned off (subject allowed to entrain) versus with the metronome turned on (not allowed to entrain). Metabolic expenditure increased by 25.8% ($p < 0.001^{*}$) when subjects walked on the treadmill wearing the harness but with no active oscillations versus when they walked on the treadmill without the harness (Baselines 2 and 1, respectively; Fig. 5a). When comparing trials with active oscillations, no significant differences were found, with the exception of one parameter combination: $\Delta f_{m} = - 6\%$ and $A_{m} = 30\% {\text{ BW}}$. This condition was more costly without the metronome compared to all other trials and baseline conditions. Still, metabolic cost did not differ significantly depending on the status of the metronome (blue vs. green in Fig. 5a) for any of the trial conditions tested. All in all, the metronome—and thus, the freedom of subjects to entrain—had no statistical effect on metabolic power. Importantly, this result did not change when controlling for the level of entrainment (e.g. ESR) in each trial condition. Furthermore, a linear mixed model found that ESR did not significantly affect metabolic power (Fig. 5b) when it was included as the primary fixed effect in a different model ($p < 0.393$). These results provide strong evidence that entrainment was not associated with reduced metabolic expenditure for subjects participating in our study.

### Subjects prefer to align peak forces after toe off

**Figure 6:**
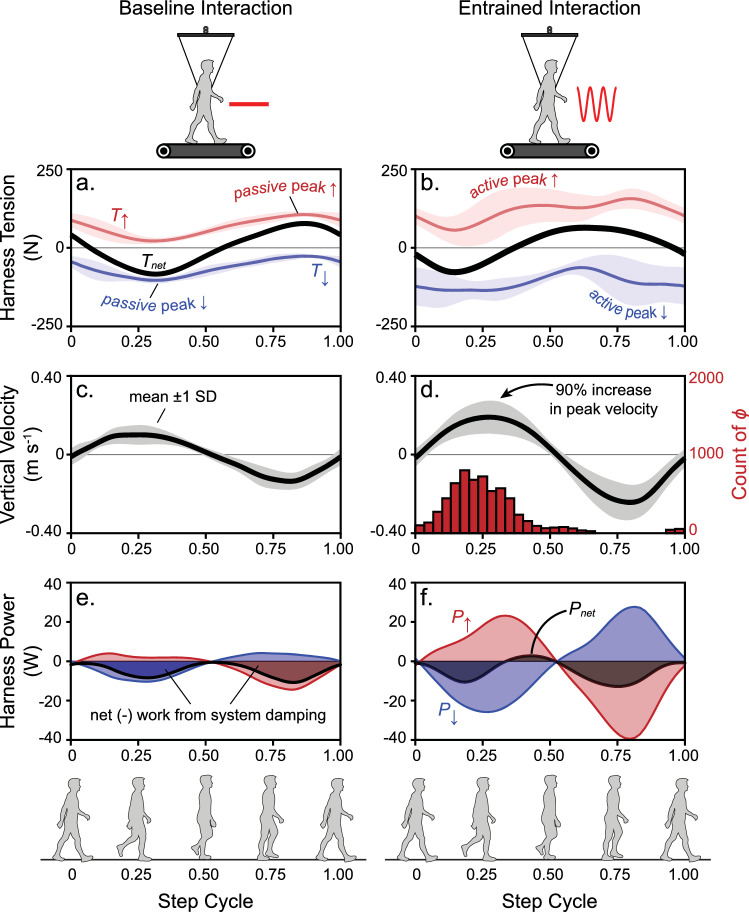
Subjects prefer to align peak oscillation forces after toe off. The average harness tension is shown in (**a**) and (**b**), where the red and blue curves indicate cable tension pulling up ($T_{ \uparrow }$) and down ($T_{ \downarrow }$), respectively, and black indicates net tension ($T_{net}$). Average center-of-mass vertical velocity is shown in (**c**) and (**d**), while the red histogram in (**d**) indicates the distribution of motor phase chosen by subjects during entrainment. Average power from the cables pulling up ($P_{ \uparrow }$, red) and down ($P_{ \downarrow }$, blue) are shown in (**e**) and (**f**), while net power ($P_{net}$) is shown in black (shading underneath the power curves represents work done by the harness forces on the person, after integration). Panels (**a**), (**c**) and (**e**) indicate mean curves (shading represents $\pm 1$ intersubject standard deviation, SD) measured during Baseline 2 (walking with the harness but no active oscillations), while panels (**b**), (**d**) and (**f**) indicate mean curves ($\pm 1$ SD) measured during experiment trials. Snapshots of the walking step cycle are shown near the bottom to help orient the reader to the timing of events shown in plots. Zero in the step cycle corresponds with double stance while 0.5 corresponds with single stance.

Figure 6a shows average tension forces measured in the harness for all subjects during Baseline 2. These forces act to pull vertically near the CoM of subjects as they walk on the treadmill. The red and blue curves indicate tension in the harness pulling up ($T_{ \uparrow }$) and down ($T_{ \downarrow }$), respectively, while the black curve is the summation of the two ($T_{net}$). Even though no active oscillations occur during the baseline test, there are still fluctuations in tension due to passive resistance of the system; these resistive forces largely occur due to the reflected inertia of the motors and associated hardware, as well as to damping effects. Given the alignment of these forces with the vertical velocity of the CoM (Fig. 6c), net negative power dominates the interaction (black curve in Fig. 6e, $P_{net}$) even as power of the cables pulling up ($P_{ \uparrow }$, red) and down ($P_{ \downarrow }$, blue) partly offset each other with both positive and negative power.

In Fig. 6b, average tension forces are shown for subjects entrained with active oscillations in the system. Although the passive force peaks observed in Baseline 2 are still present in the entrained interaction (occurs in $T_{ \uparrow }$ at around 0.80 of the step cycle), there is an additional upward peak (~ 0.35 in the step cycle) due to subjects’ preferred alignment of the active oscillations. This active force peak is further indicated by the motor phase selected by subjects during entrainment. The red histogram in Fig. 6d characterizes the distribution of motor phase chosen by subjects during entrainment, where the average and standard deviation are given: $\overline{\phi } = 0.26 \pm 0.15$. This means that peak current is prescribed to the motor pulling up just after a quarter through the step cycle (shortly after toe off of the trailing leg). This timing in the step cycle is sometimes referred to as the “rebound” phase where the body rises over the leading leg (e.g.^21^). Due to system dynamics, the current peak shows up as a spike in tension force slightly later as the “active force peak”.

The preferred motor phase seems to imply a strategy of receiving positive power from the active oscillations, given peak active force approximately aligns with peak vertical CoM velocity (Fig. 6d). Despite this alignment, negative power still greatly outweighs any positive power received from the system. Since the resistive forces largely responsible for excess negative power relate to motion of the CoM (damping relates to velocity, inertial forces to acceleration), a 90% increase in vertical velocity amplitude may help to explain why net negative power still dominates subjects during entrainment, despite a preferred phase indicating the opposite.

### Preferred phase lag varies by subject

**Figure 7:**
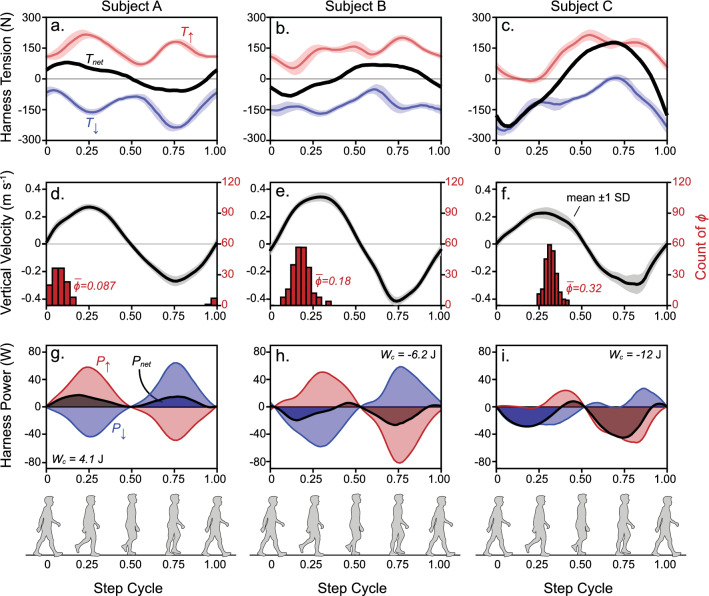
Subjects prefer a range of phase alignments, resulting in variable net power. Data from three subjects were chosen to demonstrate varying entrainment strategies. Mean data ($\pm 1$ intrasubject standard deviation, SD, in shading) for Subject A (${\Delta }f_{m} = 6\%$, $A_{m} = 30\%\;{\text{BW}}$, 297 steps averaged) are shown in (**a**), (**d**) and (**g**), where motor phase alignment occurs at $\overline{\phi } = 0.087$ of the step cycle. This strategy aligns peak tension forces with the center-of-mass (CoM) vertical velocity and thus, results in net positive work ($W_{c} = 4.1\;{\text{J}}$). Mean data ($\pm 1$ SD) for Subject B (${\Delta }f_{m} = - 6\%$, $A_{m} = 30\% \;{\text{BW}}$, 475 steps averaged) are shown in (**b**), (**e**) and (**h**) where motor phase alignment occurs at $\overline{\phi } = 0.18$ of the step cycle. Since peaks in tension due to motor forces occur slightly later, positive power is relatively low and net negative work occurs ($W_{c} = - 6.2{\text{ J}}$). Mean data ($\pm 1$ SD) for Subject C (${\Delta }f_{m} = - 6\%$, $A_{m} = 30\% \;{\text{BW}}$, 444 steps averaged) are in (**c**), (**f**) and (**i**) where $\overline{\phi } = 0.32$ of the step cycle, and mechanical power from the harness tension is dominated by resistive inertial forces, thus leading to substantial net negative work on the CoM ($W_{c} = - 12 \;{\text{J}}$). Work done on subjects by the harness forces are represented with shading underneath the power curves. Data points for subjects A, B and C are labelled in Fig. 8b.

Despite the entrainment strategy shown in Fig. 6 representing the sample mean tested in the experiment, individual subjects displayed notable variance—for example, with respect to the work done by the oscillation forces on the CoM. Figure 7 shows the average tension force, CoM vertical velocity and mechanical power of three example subjects in trial conditions chosen to illustrate entrainment strategies resulting in moderate net positive work (Subject A), moderate net negative work (Subject B) and substantial net negative work (Subject C). A notable distinction of these subjects is the chosen phase alignment of motor forces. Subject A aligned the motor phase just after zero (median phase = $31^{ \circ }$, or 0.087 in the step cycle), and distinctive tension force humps were observed shortly thereafter (~ 0.25 in the step cycle). Due to this phase, the net tension signal was locally shaped by active oscillation forces and approximately aligned with CoM velocity, thus resulting in net positive work ($W_{c} = 4.1\;{\text{J}}$). Subject B preferred a slightly increased phase ($64^{ \circ }$, or 0.18). Although the resulting motor force hump was still relatively distinct, it was not robust enough to overcome resistive forces due to inertia and damping in the system, and net negative work was accumulated over the step ($W_{c} = - 6.2\;{\text{J}}$). Subject C preferred a relatively late motor phase ($110.0^{ \circ }$, or 0.32), and forces out of phase with the CoM velocity were exaggerated. Substantial net negative work was observed in this example ($W_{c} = - 12\;{\text{J}}$). Data from example subjects in Fig. 7 imply that motor phase alignment had a prominent effect on the net mechanical work done on the CoM by the harness tension forces.

### Net mechanical work determines metabolic power

**Figure 8:**
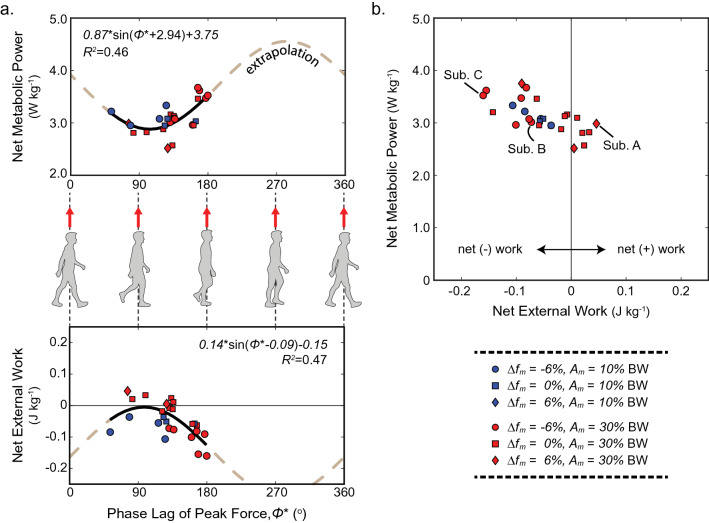
Positive mechanical work from the oscillator decreases metabolic power. Average data are shown for all trials where at least 2 min of consistent entrainment occurred (N = 27) during the time when the metronome was inactive. (**a**) Net metabolic power is plotted versus the phase lag of peak upward force from the oscillations. A phase lag of $0^{{\text{o}}}$ (or $360^{{\text{o}}}$) corresponds to peak upward force occurring at double stance while a lag of $180^{{\text{o}}}$ corresponds to peak upward force occurring at midstance (i.e. when the center of mass passes over the stance foot). Net external work done on subjects by the oscillation forces is also plotted versus the phase lag of peak upward force. Arbitrary sinusoidal functions are fit to these data assuming a cyclical relationship between center-of-mass motion and oscillation forces. (**b**) Net metabolic power decreases as a function of net mechanical work done on subjects by the oscillation forces. A linear mixed model was used to assess the effect of net work on metabolic power (both variables non-dimensionalized; see results in Table S1 in the Supplementary Materials). Example data points from subjects A, B and C are labelled for comparison in plots of Fig. 7. Blue and red data points correspond to trial conditions where $A_{m} = 10, 30\% \;{\text{BW}}$ respectively. Circles, squares and diamonds correspond to trial conditions where ${\Delta }f_{m} = - 6, 0, 6\%$ respectively.

Figure 8a shows how metabolic power and net mechanical work of the oscillations on the CoM vary due to phase lag of the active peak force pulling up. Each data point represents a trial where the subject exhibited steady entrainment and metabolic power for at least 2 min. Minimum metabolic power occurs when active oscillations pull up on the body after toe off of the trailing leg ($\phi^{*} \cong 102^{ \circ }$), and this roughly corresponds to maximum net positive work from the oscillator forces ($\phi^{*} \cong 95^{ \circ }$; Fig. 8a). Although subjects interacting with the oscillator system mostly experienced net negative mechanical work overall, variation on the gait strategy chosen illustrated a range of work done (approximately $- 0.16 {\text{ to }} {+}0.05 {\text{ W kg}}^{ - 1}$). A linear mixed model ($R^{2} = 0.74$) found a strong negative effect of net external work on metabolic cost in individual subjects and trial conditions ($p < 0.001^{*}$; see Fig. 8b). Specifically, metabolic cost was lowered by approximately 5.21 W kg^−1^ for every 1.00 J kg^−1^ increase in net work ($p < 0.001^{*}$). Conversely, the level of entrainment (i.e. ${\text{ESR}}$) was included as a covariate in the model but was not significant ($p = 0.100$). See Table S1 in the Supplementary Materials for full results of this model.

## Discussion

### Step frequency adaptations

Step frequency adaptations are comparatively large in the current study (± 6%) given levels of entrainment tend to decrease with frequencies further away from baseline^8^. However, they are still comparable to those of previous reports: approximately ± 2–8% of preferred step frequency^16–18,22^. Even so, some subjects struggled to entrain with the oscillator system even in the most favorable conditions (e.g. preferred frequency, high amplitude). Similar variability in subject response has been noted in other studies^17^. Regardless, clear trends were identified; subjects displayed the most robust and stable entrainment during trials with high motor amplitudes and frequencies below the preferred step frequency measured at baseline.

### Stronger entrainment at lower frequencies

In general, subjects exhibited higher levels of entrainment at oscillation frequencies below their preferred step frequency. Specifically, the entrainment step ratio and the average entrainment duration were both found to be negatively affected by motor frequency—meaning lower frequency conditions were associated with more consistent and robust entrainment and higher frequency conditions were associated with less consistent and robust entrainment. It is unclear why stronger entrainment was associated with lower oscillation frequencies. While one study showed some evidence of increased entrainment at external oscillation frequencies lower than baseline step frequencies^4^, others have not reported similar asymmetries related to step frequency adaptations^16^.

### Metabolic cost of oscillator interaction strategies

Variation in metabolic power was not affected by entrainment but was instead strongly related to net mechanical work done on the subject by the harness tension. Specifically, receiving net positive work was associated with a lower energetic cost than with negative work. This result may be reasonable given: (1) zero net CoM work is required for steady, periodic gait (e.g. net positive oscillator work requires net negative muscle work, and vice versa) and (2) negative muscle work costs 4.8 times less metabolic energy than positive muscle work^23^. This logic assumes that no additional work is needed to compensate for extra force on the body, other than the net negative work needed to manage the energy balance. It is unclear if this assumption holds.

Ideally, the subject learns a strategy to receive positive power from the oscillator system in such a way that the leg muscles are unburdened from their typical function (e.g.^10,24^). Indeed, large amounts of positive mechanical power naturally occur due to push off forces during the step-to-step transition in the gait cycle, functioning to redirect the body from falling to rising with the next step^25^. Recent optimization models have identified a strategy for minimizing work while walking with an oscillating impulse applied to the CoM^26^. This strategy aligns upward oscillator forces with the step-to-step transition (phase = $0^{ \circ }$). The upward oscillator force does some work that the legs would normally do, thus minimizing cost in the model. Notably, this is a different strategy than what subjects preferred in the current study, where individuals roughly aligned oscillation forces with CoM vertical velocity to maximize externally applied positive power (phase $\approx 130^{ \circ }$, Fig. 8a). However, the model did not account for differential energetic costs of positive and negative muscle work, nor did it allow for negative work via passive dissipative mechanisms (e.g. collisions during foot–ground contact^27–30^, hysteresis in soft tissue deformation^31,32^, damping-like forces from the oscillator system, etc.) which require near zero metabolic expenditure. Furthermore, the model did not account for swing leg dynamics and associated costs, nor elastic properties of the legs. These issues and others may be important for understanding subject preferences of increased positive mechanical power from the oscillations.

If the preferred strategy uses oscillation forces to replace positive muscle work, then the extent to which this strategy is energetically favorable likely relies on an individual downregulating leg work and associated muscle activity^24^. Yet subjects substantially increased the vertical excursion of their CoM when they entrained to motor oscillations compared to at baseline (an increase of 58.2% or 125.5% for $A_{m} = 10\%$ or $30\% {\text{ BW}}$, respectively). The increased body oscillation is likely evidence that subjects did not downregulate their push off during entrainment. Instead, it seems that subjects preferred to increase positive power from the system by aligning active force peaks somewhat after toe off in the step cycle (e.g. Figs. 6b, 8a). Furthermore, subjects that leveraged the most positive power from the oscillations had the lowest metabolic cost (local minimum at $\phi^{*} \cong 102^{ \circ }$). These results, however, should be treated with caution. Experiments presented here did not explicitly control or manipulate the motor phase, but rather, the phase variation depicted in Fig. 8a represents subject preferences during the experiment with no metronome. Since subjects did not explore a full range of phase values in steady state, it is unclear if the preferred alignment centers on a global or local optimum. The brown dashed line in Fig. 8a indicates a sine wave fit to the data with a least squares regression, assuming a cyclical cost over phase. Extrapolation of this fit beyond the relatively narrow range of data is questionable, and future studies could control motor phase explicitly to characterize fluctuations in cost.

Notably, the data show mostly net negative work in the sampled phase domain, despite maximal positive power expected in this region ($\phi^{*} \cong 95^{ \circ }$). This bias may partially be explained by negative work associated with resistive forces due to reflected inertia of the motors and hardware, as well as damping in the system (e.g. back electromotive force). Increases in vertical excursion of the CoM during entrainment likely contributed to an exaggeration of these effects. Perhaps subjects prefer phase lags that maximize positive mechanical power from the oscillations in part to offset dissipation in the system.

### Interactions with other active devices

It is possible that the motor control system uses mechanical and/or physiological variables as a proxy for energetic cost, as other researchers have previously suggested^33^. In this case, it appears that subjects prefer to maximize positive mechanical power from the oscillations. However, it is unclear if this preference is generalizable to mechanical interactions with other devices or if it is only relevant to the specific system discussed here. Ahn and Hogan^10,11^ found that subjects aligned ankle torques at push off with those from an ankle exoskeleton. Since the ankle generates high positive power during push off, the alignment likely means that individuals chose to leverage positive power from the device, similar to our subjects. A different study found that individuals prefer to align pulses of electrical stimulation to the plantar flexors either just before toe off or in advance of heel strike^6^.

Experiments by Selinger et al^16^ found subjects adjust their gait in response to resistive damping forces from a knee exoskeleton. While subjects could not possibly receive positive power from the device, they did actively adjust their gait to avoid negative power. Sánchez et al^34^ investigated gait adaptation of subjects walking on a split-belt treadmill (a treadmill that contains separate belts moving at different speeds for each leg). The authors showed that when subjects are given sufficient time adapting to the system, they employ a step length asymmetry associated with net positive mechanical power from the treadmill and reduced metabolic output.

These examples provide some evidence of preferred subject interactions that involve either reducing net negative work or increasing net positive work from dynamic external devices. This strategy is consistent with our results that subjects prefer to align motor forces approximately with vertical CoM velocity. However, it is unclear if the ultimate objective is to increase positive power or to decrease metabolic expenditure, since the two are often correlated. Wong et al^35^ showed that individuals do not adjust gait in exchange for higher levels of oxygen concentration fed to them through an air tube, even as they consciously acknowledge an increased effort from not adjusting their gait. Perhaps individuals are sensitive to positive mechanical power from external sources as an indirect cue of economical interactions with the environment.

### Entrainment may stabilize interactions for internal models of gait

Given that most subjects learned to entrain under a broad range of oscillation parameters, it seems reasonable to conclude that individuals largely preferred to stabilize interactions with their environment. Here, stability does not necessarily refer to fall avoidance or balance, but rather to a state of relative consistency, where interactions with the environment are sufficiently repeatable over subsequent steps. The preference for a stabilized environment could be interpreted as evidence of a feedforward gait control mechanism since unpredictability could impede effective implementation of internal models. Various studies have shown evidence of a dual-part locomotor control process, including a rapid response to external stimuli and a slower, more gradual, fine-tuning of the response^33,36,37^. These findings are interpreted as evidence of an internal model used to make quick predictions (within seconds) regarding energetic cost based on state estimations and followed up with direct energy optimization occurring more slowly (within minutes). Other researchers have proposed that feedforward and feedback control mechanisms also play a role during gait adaptation to split-belt treadmills^38^ and lateral perturbation systems used to train individuals with incomplete spinal cord injury^39^. The experiments presented in the current study describe subject interactions that can be relatively volatile, at least before subjects converge on entrainment. In particular, inexperience with the oscillation system may require a prerequisite to the dual-part control of locomotion described previous: a stabilization phase. A relatively stable interaction could foster successful implementation of feedforward or feedback control, and entrainment could provide that stability.

Koban et al^40^ explained a similar perspective in a slightly different context, with regards to individuals that entrain gait when walking side by side (i.e. interpersonal synchronization). They described a process by which entrainment occurs to reduce the perceived mismatch between an expectation about their companion’s motor behavior based on their own. An analogous principle could be adapted to the expectation of mechanical interactions with the environment, as mediated by the entrainment opportunities associated with the oscillator system described in this manuscript. In lieu of a direct metabolic motivation for entrainment, it may be possible that the motor control system prefers a relatively stable interaction with the environment so as to make feedforward predictions more precise and actionable.

Indeed, we attempted to control for various aural and visual stimuli (i.e. stimuli other than dynamical interactions with external oscillations) that may have influenced subject entrainment. In the same way that people often match the actions of companions in close coordination^41–43^, it is possible that subjects would have entrained with the visual cues of the motors or pulleys bobbing vertically in time, if they had access to them. Similarly, if not for the use of headphones to block out audio cues, the rhythmic sounds of the system’s oscillation could potentially provide a coherent tempo to match steps with, similar to how individuals adjust running cadence to musical tempos during exercise^8,9^. Instead, subjects likely had access to tactile information through tension in the harness, as well as to changes in loading on the body via biosensors such as Golgi tendon organs. While subject entrainment is helpful for stabilizing interactions with the environment, it remains unclear if and to what degree information from various stimuli are differentially prioritized and if such considerations are task dependent.

### Potential practical applications

Insights from the current study could be useful when designing gait assistive machines or rehabilitation protocols. For example, previous and current research on exoskeleton design has been limited by understanding what users need from their assistive devices (e.g. power, torque, etc.). An injection of positive power can reduce energetic cost in optimization protocols where parameters of ankle torque assistance are customized for the individual^44^. However, the same study showed subjects reduce power from the exoskeleton when they adapt joint kinematics to a static torque assistance profile over time^44^. While both of these observations seem to conflict with one another, it is possible that individuals prefer to avoid excessive power at specific joints whilst simultaneously benefitting energetically from positive power at the whole-body level. This interpretation is supported by results in our current study, where subjects selected phase relationships that maximize positive external power and reduce energetic cost.

Entrainment as a method for securing stability in interactions with the environment can also be exploited to improve training protocols with assistive devices or rehabilitation. For example, various metrics have been proposed to assess the stability of coupled-oscillator interactions^43,45,46^ and could be provided to users via visual feedback in order to fast-track training. Perhaps the simplest metric is the standard deviation of relative phase between the human and the machine. This metric assesses the distribution of relative phase and thus, describes stability of the interaction. Another previously proposed metric is cross-spectral coherence, which can be used to analyze the level of coordination between assistive machines and their users across relevant frequencies. Any of these metrics could potentially be used to provide visual feedback in order to accelerate efficient interactions with artificial systems. Alternatively, control systems could be designed to eliminate drift in relative phase of relevant interactions.

## Limitations and future work

There are a few limitations to this work which should be carefully considered. For example, our experiments were designed to give subjects a limited exposure to external oscillations in order to accommodate testing of multiple oscillation/experimental parameters: e.g., two amplitudes, three frequencies, metronome constraint, baseline conditions, etc. As such, each subject was only given 5 min per condition to learn entrainment with the system. It is certainly possible and perhaps likely that we would have found much higher levels of entrainment and reduced cost with entrainment if subjects were given more time to acclimate. Indeed, recent studies have argued individuals may require well over 20 min exposure time before they are well-adapted to novel tasks^34^. Furthermore, relatively small sample size may limit the generalizability of entrainment responses measured here.

Figure 8a seems to imply that subjects with relatively stable interactions approximately chose phase relationships that minimized energetic cost. However, these data represent natural variation in experiments, and the results should be treated with caution. Future work should control phase of the oscillation forces in order to more carefully measure the energetic landscape.

It is also possible that entrainment can be explained through details of the experimental implementation not related to the dynamics of the human–machine interaction. For example, entrainment may be affected by comfort levels associated with the harness rubbing on the individual’s body, due to an imperfect attachment. Individuals might also target their steps, due to size limitations of the treadmill. However, this seems somewhat unlikely since subjects had higher levels of entrainment at lower frequencies even though this would require longer steps (i.e. less space to maneuver on the treadmill).

Another limitation is the implementation of oscillation force control in the mechatronics system. The controller was designed as an open loop current control, in part for simplicity, and thus, neglects any compliance or dissipation in the pulley system or harness. Future designs could be improved with the implementation of a closed loop force control in order to ensure force oscillations are felt by the subject precisely as prescribed. More generally, it is unclear if the entrainment results found here are specific to the precise task, or if they apply more broadly to circumstances where periodic environmental oscillations occur. Furthermore, we only tested entrainment behaviors at a single, moderate walking speed. It is unclear how our results might differ with walking speed. Future studies should test the effect of gait type or locomotion speed on entrainment behaviors.

## Conclusions

The energetics of entrainment was evaluated in a human–machine coupled oscillator system. Specifically, a novel mechatronics oscillator system was used to vary vertical tension periodically in a body harness worn by subjects walking on a treadmill. Subjects entrained with the system over a wide range of oscillation parameters (amplitude, frequency), but in particular, at high amplitudes and frequencies lower than baseline preferred step frequencies. While energetic cost was not significantly affected by the level of entrainment in experiments, subjects who had net positive work done on them by the external oscillations had a lower cost than those with net negative work. Net work was largely determined by the alignment of oscillation forces within the step cycle, and positive work was maximized when subjects aligned peak upward force after toe off from the trailing leg. We interpreted entrainment as a means for subjects to seek stable interactions with the environment in order to apply effective locomotor control in a novel task. These results appear to highlight an underappreciated aspect of walking control and can help to inform the development of machine and robotic interfaces for applications such as prostheses or other gait assistive devices.

## Methods

### Participants

A convenience sample of ten healthy university students (five males, five females) were recruited. The mean (± 1 standard deviation, SD) subject height was 1.71 ± 0.07 m, leg length (measured as the distance from the ground to the greater trochanter while standing) was 0.91 ± 0.06 m, weight was 65.7 ± 12.2 kg and age was 26.2 ± 2.9 years. Exclusion criteria for the experiments included any musculoskeletal injuries or neurological conditions affecting one’s gait or their ability to carry a heavy backpack. All participants provided informed consent to participate, and these studies were approved by an ethics review board at the University of Calgary (REB16-1517). In addition, informed consent was obtained to display images of the participant shown in Fig. 1b. All methods were performed in accordance with the relevant guidelines and regulations.

### Measurements and analysis

Inertial measurement units, or IMUs (Xsens Technologies B.V., Enschede, The Netherlands) were placed at each ankle and the lower back. The ankle sensors were used to detect signal peaks and calculate step frequency as the inverse of the time period between peaks. The back sensor provided kinematics to approximate CoM motion (acquisition rate = 100 Hz). These data were integrated twice over time to get velocity and displacement, and a moving average (window set to stride time) was subtracted from the signal to adjust for low frequency drift error, assuming steady, periodic gait and thus, net zero velocity/position with each stride. This approach is similar to validated procedures that adjust for drift by subtracting error at known zero-velocity points in the stride cycle^47^. A moving average filter (window of ± 5 steps at each data point) was also applied to step frequency measurements to more clearly illustrate trends over time. Step frequency ($f_{s}$) was normalized by the subject’s baseline frequency ($f_{p}$) measured while walking in the system with the motors inactive.

1 $$f_{r} = \frac{{f_{s} }}{{f_{p} }}$$

Custom in-line tension transducers were built with strain gauges (Micro-Measurements CEA-06-125UW-350, Wendell, NC, USA) configured in half-bridge circuits. The strain gauges were epoxied to C-shaped steel hooks and measured tension in cables pulling on the body harness during experiments (Fig. 1). The strain gauge signals were passed to a strain conditioning amplifier (National Instruments, SCXI-1000 with SCXI-1520 eight-channel universal strain gauge module connected with SCXI-1314 terminal block, Austin, Texas USA), digitized (NI-USB-6251 mass termination) and acquired in a custom virtual instrument in LabVIEW (National Instruments) at an acquisition rate of 100 Hz. The transducers were calibrated with known weights before every testing session using a least squares linear regression to quantify a conversion factor from volts to Newtons force ($R^{2} > 0.99$).

At the beginning of each trial, a few seconds of data were collected where the subject stood still before starting the treadmill. During this time, the transducer signals measured force from the motors’ weight in the system plus nominal tension from the motors pulling the system taut. This initial tension was averaged over a 10 s interval and subtracted from the subsequent signal in the trial.

Next, tension forces were multiplied by vertical velocity to calculate mechanical power acting on the CoM. Step cycles were distinguished by peaks in the vertical acceleration of the CoM (approximately indicating the middle of double stance). Tension forces, kinematics and mechanical power were all segmented into blocks of data comprising every step cycle identified in all trials. These data were interpolated at regular intervals matching the average resolution of the raw data collection (~ 55 data points per step).

Pulse signals were recorded in LabVIEW to mark the timing of peak current sent to the motors relative to the start time of a given step cycle ($\Delta t_{p}$). In addition to data synchronization, the pulse signals were used to calculate the phase lag of peak current relative to the step cycle ($\phi$).

2 $$\phi = \Delta t_{p} f_{s} \times 360^{\circ}$$

Due to dynamics of the system, there was a slight delay from when peak current was driven to the motors to when tension spiked in the harness. The average delay, $\Delta \phi$, was calculated for all subjects and trials and phase data were shifted as appropriate: $\phi^{*} = \phi + \Delta \phi$.

Oxygen consumption and carbon dioxide elimination rates were measured using a commercial metabolic analysis system (TrueMax 2400, ParvoMedics, Salt Lake City, UT, USA). Experiments were divided into multiple phases (e.g. motors active, metronome inactive; motors active, metronome active; see Fig. 2a) over which, metabolic data were collected continuously. Each phase lasted 5 min, allowing metabolic data to reach steady state conditions before mean and SD values were calculated (typically the remaining 2–3 min of each phase). Oxygen consumption rates in ${\text{ml}}\;{\text{O}}_{2} \;{\text{s}}^{ - 1}$ were multiplied by a factor of 20.1 to convert to Watts, assuming a mixed diet of fat and carbohydrates^48–50^. Gross metabolic rate was converted to net metabolic rate by subtracting baseline values during quiet standing. Net metabolic power was non-dimensionalized by dividing belt speed and subject BW (sometimes referred to as non-dimensional cost of transport). During all metabolics testing, the data were deemed acceptable if the respiratory exchange ratio remained below a value of 1.0.

### Test protocol

During Baseline 1, subjects walked on the treadmill freely (i.e. without the body harness; Fig. 2b) for a 5-min duration. The treadmill speed ($v_{b} = 1.19\;{\text{m }}\;{\text{s}}^{ - 1}$ on average) was programmed such that non-dimensional speed, $\tilde{v}$, equaled 0.4 during all testing.

3 $$v_{b} = \tilde{v}\sqrt {gL}$$

where $L$ is the subject’s leg length. Speed was rounded to the nearest tenth of a km/h, per the treadmill’s available resolution.

During Baseline 2, subjects walked while wearing the body harness connected to the pulley-cable system (Fig. 2b) for a 10-min duration. Both actuators provided a constant nominal tension (approximately 10% BW) to reduce slack in the system, but the average net force on the body was zero since one cable pulled up while the others pulled down. Subjects also experienced added inertia from the motors and associated hardware connected in the system, as well as friction and damping. Each subject’s baseline preferred step frequency ($f_{p}$) was assessed at treadmill speeds used in the experiments and motor frequencies prescribed in testing conditions were determined relative to this baseline.

During experiments, subjects walked in the harness for 2 min with nominal motor current to avoid cable slack. Next, current oscillations were commanded to the motors at a constant frequency and amplitude, and the subject was instructed to respond freely (Fig. 2a). Five minutes later, subjects were asked to step to the beep of a metronome (matched to their baseline frequency, i.e. “frequency clamping”; Fig. 2a) even as oscillations continued at a different frequency. After another 5 min, the oscillations and metronome ceased, and the subject prepared to end the trial. Metabolic data were collected throughout this test to compare oxygen consumption while responding freely in the system (allowed to entrain) versus walking to the metronome (not allowed to entrain). This test was performed with various oscillation amplitudes ($A_{m} = 10, 30\% {\text{ BW}}$) and motor frequencies relative to baseline step frequency ($\Delta f_{m} = 0, \pm 6\%$; see Fig. 2b).

4 $$\Delta f_{m} = \frac{{f_{m} - f_{p} }}{{f_{p} }} \times 100\%$$

The amplitudes and frequencies used in experiments were selected based on early pilot data demonstrating a sufficient level of entrainment at the values chosen. In the case of trial conditions where $\Delta f_{m} = 0\%$, no metronome was played and the trial ended after 5 min of oscillations. Trial conditions were randomized to minimize any ordering effects.

During experiments, a curtain was used to blind subjects from any motion of the pulleys or motors (Fig. 1b). Ambient noise was played through headphones to help block out rhythmic sounds of the system. During trials, subjects were asked to walk in any manner that felt most natural or extracted minimal effort. However, subjects were encouraged to explore different aspects of their gait, including step length. Note, additional details describing the oscillator system design and operation can be found in the Supplementary Materials.

### Defining entrainment

Entrainment was defined with arbitrary thresholds: any step frequencies measured within ± 3 SDs (~ ± 0.02 Hz or about 1%^8^ of the average step frequency chosen during preferred baseline conditions) of the prescribed motor frequency, where the SD was determined from the last minute of data in Baseline 2 (treadmill walking with the harness). Two metrics quantified the level of entrainment for a subject in each trial. The entrainment step ratio (ESR) is the ratio of entrained steps to total steps taken during experiment (without the metronome) while the average duration of entrainment ($\Delta \overline{t}_{e}$) was used to estimate entrainment durations since subjects sometimes drifted in and out of the motor’s frequency. The average entrainment duration was calculated as: ${\Delta }\overline{t}_{e} = \frac{1}{N}\mathop \sum \nolimits_{i = 1}^{N} \frac{{{\Delta }t_{e,i} }}{300}$, where $N$ is the number of bouts of entrainment in a trial and ${\Delta }t_{e,i}$ is the time duration of each individual entrainment bout, in seconds (see Fig. 4). Furthermore, a sensitivity analysis was conducted to determine how the level of entrainment per oscillation amplitude and frequency is affected by the choice of entrainment thresholds (see Supplementary Materials for these results).

### Statistical analysis

Filtered relative step frequency data were interpolated at equal time intervals matching the data acquisition rate. Median values of the interpolated data were taken across all subjects who entrained at least once in the trial and quartiles characterized the spread of the distribution for each time point at 25% and 75% levels. Quartiles were used (as opposed to standard deviations) to better represent skewed distributions of the data (see shaded error in Fig. 3 for examples).

Linear mixed models were used to assess various outcomes during experiments. The mixed model was chosen to control for repeated measurements among subjects participating in multiple trials each; subject was included in the models as a random effect. All statistical models were developed and evaluated in JMP (SAS Institute Inc., Cary, NC USA, version 14.1.0) using the restricted maximum likelihood method for parameter estimation and a compound symmetric covariance structure.

In four models, motor frequency (${\Delta }f_{m}$) and amplitude ($A_{m}$) as well as an interaction between the two (${\Delta }f_{m} \times A_{m}$) were added as fixed effects to test if the oscillation parameters contributed significantly to the various outcomes. The first two models tested the effect of oscillation parameters on the level of entrainment via ESR and ${\Delta }\overline{t}_{e}$.

Metabolic measurements were statistically compared depending on the prescribed oscillation parameters (frequency and amplitude), baseline type (wearing or not wearing the harness) and the metronome’s status (i.e. active or inactive) during data collection. A post hoc Tukey’s Honestly Significant Difference test was used to detect differences in estimates of metabolic power ($\alpha = 0.05$), while controlling for multiple hypothesis testing. Given the metronome’s status only indicates whether an individual has the freedom to entrain and not whether they actually did entrain, a separate model was used to test for the effect of entrainment level (via entrainment step ratio, ESR) on metabolic power. ${\Delta }\overline{t}_{e}$ was not included to avoid collinearity. A final model also included net mechanical work done by the harness tension (in addition to fixed effects described previously) to assess any effect of the mechanical interaction on cost.

The significance of fixed model effects was evaluated with 95% confidence limits and post hoc *t* tests where *p* values were adjusted ($p_{adj}$) using the Bonferroni correction depending on how many tests were performed in the model. Tests were considered significant if $p_{adj} < 0.05$. Throughout the manuscript, unadjusted *p* values are reported, and significance is indicated with asterisks. A summary of statistical model results is found in the Supplementary Materials.

## Supplementary Information

Supplementary Information.
