# Supplementary material for: Evaluating the energetics of entrainment in a human–machine coupled oscillator system
Source: Sci Rep. 2021 Aug 4;11:15804. doi: 10.1038/s41598-021-95047-x (PMC8338938; doi:10.1038/s41598-021-95047-x)
Supplement: Supplementary file 1 — Supplementary Information. [file 41598_2021_95047_MOESM1_ESM.pdf]

Supplementary Materials for "Evaluating the energetics of entrainment in a human-machine coupled oscillator system"

Ryan T. Schroeder[1,2,3,4], James L. Croft[3], and John E.A. Bertram[1,2,4]

[1]Biomedical Engineering, University of Calgary, Calgary, AB, Canada

[2]School of Medical and Health Sciences, Edith Cowan University, Perth, WA, Australia

[3]Faculty of Kinesiology, University of Calgary, Calgary, AB, Canada

[4]McCaig Institute for Bone and Joint Health, Cumming School of Medicine, University of Calgary, Calgary, AB, Canada

This document contains additional details regarding the design and operation of the oscillator system used in experiments described in the main text. Additional analysis and full statistical results are also included.

Machine oscillator system

General design

We developed a custom pulley-cable system (see Fig. 1 from main text) that connects subjects wearing a body harness with two linear servomotors (Nippon Pulse America Inc., Radford, Virginia USA; model: S320T). Each actuator was activated to create varying tension in the system (one pulling up on the individual, one pulling down). The harness tension acted as a periodic perturbation applied to subjects walking on a treadmill. The cables pulling downward were connected to the body harness near the waist at oblique angles in the frontal plane (approximately 75˚ from horizontal, depending on the waist height of the subject; Fig. 1b from main text). Thus, horizontal components of the tension vectors largely cancelled out; any net mediolateral forces due to asymmetry were neglected. All three cables (one pulling up, two pulling down obliquely) were redirected via pulleys mounted on carts that could roll in the fore-aft direction so the subject could drift slightly on the treadmill without altering the loading direction.

Even when the motors were inactive, subjects experienced inertial resistance from the mass of the motors and hardware attached to the cables (approximately 2.2 kg or 3.3% of an average subject's body mass). The motors were mounted to aluminum plates supported by ball-bearing linear guides (Chieftek Precision Co., LTD., Chino, California USA) to stabilize uniaxial motion. The actuators were controlled by a duel-axis motion controller (Galil Motion Control, Inc., Rocklin, California, USA; model: DMC-4123)

with sinusoidal amplifiers (model: D3520). Additionally, a direct current power supply (Advanced Motion Controls, Camarillo, California, USA; model PS16L80) was used to power the actuators.

Motor current control

The controller prescribed a sinusoidal current signal, $I(t)$, to the motors as desired.

$$I(t) = I_m \cos(\omega_m t) \quad \text{(S1)}$$

where I_m is the current amplitude, ω_m is the motor frequency ($rad\ s^{-1}$) and t is time. The commanded signal was distributed between both motors – one pulling up, one pulling down. For example, when a positive current was commanded, the sinusoidal signal was prescribed to the motor pulling upward ($I_\uparrow$) while a constant current (I_{nom}) was prescribed to the motor pulling downward ($I_\downarrow$). This maintained nominal tension in the cables and harness. When a downward force was desired, the motors switched roles.

$$I(t) = I_\uparrow(t) - I_\downarrow(t) \quad \text{(S2)}$$

$$I_\uparrow(t) = \begin{cases} I_m \cos(\omega_m t) + I_{nom} & , \quad I(t) \geq 0 \\ I_{nom} & , \quad I(t) < 0 \end{cases} \quad \text{(S3)}$$

$$I_\downarrow(t) = \begin{cases} I_{nom} & , \quad I(t) \geq 0 \\ I_m \cos(\omega_m t) + I_{nom} & , \quad I(t) < 0 \end{cases} \quad \text{(S4)}$$

Amplitude (A_m), prescribed in units of body weight force (BW), was converted to Amperes of current by multiplying the subject's BW, dividing the motor force constant (K_f) and dividing the gearing ratio (R) of the pulley-cable system.

$$I_m = \frac{A_m M g}{R K_f} \quad \text{(S5)}$$

where M is the subject's body mass, g is the gravitational acceleration constant (9.81 $m\ s^{-2}$). The desired current signal was prescribed with an open loop control. Force feedback was not implemented for simplicity; however, tension in the pulley-cable system was monitored throughout all experiments.

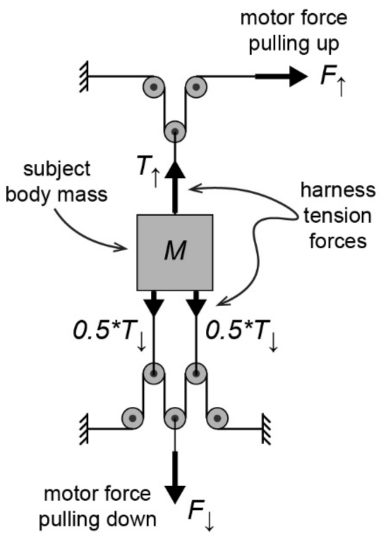

Figure S1. Pulley-cable system diagram. Configurations are shown for both pulley-cable sections (pulling up and pulling down). All inertia of the pulley-cable system and the motors is neglected, and contact surfaces are assumed frictionless. Furthermore, the cables are assumed rigid and always in tension.

The pulley-cable system is configured into two sections: one pulling upward and one pulling downward on the body harness worn by subjects (Fig. S1). All inertia associated with the pulleys, the cables and the motors are neglected. Additionally, friction and compliance are not considered in the simple analysis. Motor force in the upward section ($F_\uparrow$) is related to the tension ($T_\uparrow$) in the cable directly pulling on the body.

$$T_\uparrow = 2F_\uparrow \quad \text{(S6)}$$

Where motor force is assumed to vary proportional to motor current.

$$F_\uparrow = I_m K_f \quad \text{(S7)}$$

Peak tension was matched to the desired oscillation amplitude given the following equation:

$$T_\uparrow = A_m M g \quad \text{(S8)}$$

Eqs. (S6-S8) are combined to determine the current amplitude required per desired motor amplitude in Eq. (S5), where $R = 2$ is the gearing ratio relating tension force applied to the body harness with the motor force. In the downward section, motor force ($F_\downarrow$) is related to the tension ($T_\downarrow$) in the cable pulling on the body via the same gearing ratio as the upward section, i.e. $T_\downarrow = 2F_\downarrow$. Thus, Eq. (S5) also applies to the motor pulling downward with the same gearing ratio.

Entrainment threshold sensitivity analysis

During analysis, subject entrainment was defined by instances where step frequency was measured within a small range above and below the oscillation frequency used in each trial. This entrainment threshold was applied as multiples of standard deviations (SD) calculated from the last minute of subject step frequency data during Baseline 2 (treadmill walking with the harness). Specifically, ±3 SDs (~±0.02 Hz or about 1% of the average preferred baseline step frequency) was used for the threshold during analysis.

Since the choice of this threshold was somewhat arbitrary, a sensitivity analysis was conducted to show the effect of varying the threshold range. In Fig. S2a, step frequency data are shown for an example trial where transient data occurred (same trial from Fig. 4a in the main manuscript). The colored bands (width indicated with closing arrows) correspond to three different entrainment thresholds (±1, ±3 and ±5 SDs). As such, any step frequency data occurring within a given threshold indicates entrainment. The entrainment step ratio (ESR) was calculated for all trials and the three entrainment thresholds to determine any effect on the average trend for all subjects. As shown in the main manuscript (Fig. 4c), the ESR increases with amplitude and decreases at higher frequencies. Unsurprisingly, a smaller entrainment threshold results in a somewhat lower ESR. However, the general trends are not greatly affected as a function of the oscillation amplitude or frequency.

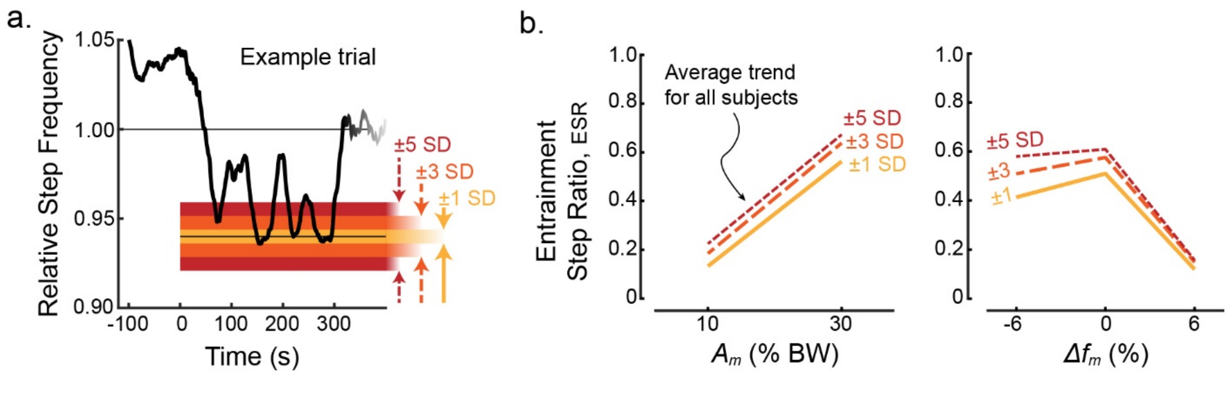

Figure S2. Entrainment threshold sensitivity analysis. (a) An example trial shows transient entrainment depending on the entrainment thresholds shown as light orange, orange and dark red bands (±1, ±3 and ±5 standard deviations, or SDs, respectively). The subject is considered to be entrained when step frequency measurements occur within the band of a given entrainment threshold. (b) Entrainment Step Ratio, or ESR, is shown for the average of all subjects walking with oscillations of various amplitudes and

frequencies (A_m and Δf_m, respectively). The trendlines correspond to the colored bands show in the example trial, depending on the entrainment threshold.

Full results of statistical models

Linear mixed models were used to assess the effect of oscillation parameters on various outcomes including the level of entrainment and metabolic power (Table S1). Subject was included in all the models as a random effect. The significance level was set to $\alpha = 0.05$ and *p* values were adjusted with the Bonferroni method depending on the number of tests performed in each model. All statistical models were developed and evaluated in JMP (SAS Institute Inc., Cary, NC USA, version 14.1.0) using the restricted maximum likelihood method for parameter estimation and a compound symmetric covariance structure. The entrainment step ratio (ESR) and net mechanical work (W_c) from the oscillator were added as covariates in Models 3.) and 4.) respectively, where the effect of oscillation parameters on metabolic power was also assessed.

An additional linear mixed model was run with oscillation parameters, baseline type (harness worn or not worn) and metronome status (active or inactive) as fixed effects in order to evaluate metabolic power in multiple testing conditions. A *post hoc* Tukey's Honestly Significant Difference (HSD) test was applied to detect statistical differences in all metabolic conditions measured in experiments (Table S2).

Table S1. Summary of statistical models on experiment data.

Model Description	Dependent Variable	Independent Variables	β	Lower CL	Upper CL	*p*
1.) Entrainment step ratio versus trial conditions	ESR	Intercept	-0.042	-0.230	0.146	0.656
		Δf_m	-3.005	-4.607	-1.402	<0.001*
		A_m	2.267	1.482	3.052	<0.001*
		$\Delta f_m \; x \; A_m$	-8.838	-24.862	7.185	0.273
2.) Average time duration of entrainment versus trial conditions	$\Delta \bar{t}_e$	Intercept	-0.030	-0.227	0.166	0.756
		Δf_m	-2.712	-4.302	-1.121	0.001*
		A_m	1.737	0.958	2.517	<0.001*
		$\Delta f_{\mathrm{m}} \; x \; A_m$	-12.269	-28.174	3.637	0.127
3.) Metabolic cost of transport versus level of entrainment	CoT_{met}	Intercept	0.257	0.238	0.276	<0.001*
		Δf_m	-0.102	-0.171	-0.033	0.004*
		A_m	0.040	0.001	0.078	0.047
		$\Delta f_m \; x \; A_m$	-1.019	-1.644	-0.395	0.001*
		ESR	-0.005	-0.015	0.006	0.393
4.) Metabolic power versus net mechanical work	P_{met}	Intercept	0.106	0.086	0.127	<0.001*
		Δf_m	0.070	-0.045	0.185	0.218
		A_m	0.030	-0.009	0.069	0.121
		$\Delta f_m \; x \; A_m$	-0.026	-1.403	1.351	0.968
		ESR	-0.018	-0.041	0.004	0.100
		W_c	-1.587	-2.271	-0.902	<0.001*

Summary of linear mixed models used in the manuscript. These models tested the effect of motor frequency (Δf_m as a fraction of preferred step frequency during baseline), amplitude (A_m as a fraction of body weight) and an interaction between the two ($\Delta f_m \; x \; A_m$) on the entrainment step ratio (ESR), the average time duration of entrainment ($\Delta \bar{t}_e$), metabolic cost of transport (CoT_{met}) and metabolic power (P_{met}). The ESR was added to the third model to assess whether the level of entrainment determined energetic cost. Finally, net work done on the subject by the oscillations (W_c) was added to the fourth model as a covariate to evaluate the mechanical interaction on cost. All variables are non-dimensionalized with combinations of M, g and L. Confidence limits (CL) are set to a level of 95% and *p* values are marked with an asterisk to indicate significance after controlling for multiple testing.

Table S2. Summary of Tukey's HSD on metabolic power data during experiments.

Level 1 (L1)				Level 2 (L2)							
Δf_m	A_m	Met.	Exp. Type	Δf_m	A_m	Met.	Exp. Type	L1-L2	Lower CL	Upper CL	*p*
-6	30	off	CoE	0	0	off	BL1	0.089	0.071	0.107	<0.001*
-6	30	on	CoE	0	0	off	BL1	0.075	0.056	0.094	<0.001*
0	10	off	CoE	0	0	off	BL1	0.067	0.049	0.085	<0.001*
6	30	on	CoE	0	0	off	BL1	0.063	0.044	0.082	<0.001*
6	30	off	CoE	0	0	off	BL1	0.062	0.044	0.080	<0.001*
6	10	off	CoE	0	0	off	BL1	0.061	0.043	0.079	<0.001*
6	10	on	CoE	0	0	off	BL1	0.061	0.043	0.079	<0.001*
-6	10	off	CoE	0	0	off	BL1	0.061	0.043	0.078	<0.001*
0	30	off	CoE	0	0	off	BL1	0.060	0.042	0.078	<0.001*
-6	10	on	CoE	0	0	off	BL1	0.060	0.042	0.078	<0.001*
0	0	off	BL2	0	0	off	BL1	0.051	0.033	0.069	<0.001*
-6	30	off	CoE	0	0	off	BL2	0.038	0.020	0.056	<0.001*
-6	30	off	CoE	-6	10	on	CoE	0.029	0.011	0.046	<0.001*
-6	30	off	CoE	0	30	off	CoE	0.029	0.011	0.046	<0.001*
-6	30	off	CoE	-6	10	off	CoE	0.028	0.011	0.046	<0.001*
-6	30	off	CoE	6	10	on	CoE	0.028	0.010	0.045	<0.001*
-6	30	off	CoE	6	10	off	CoE	0.028	0.010	0.045	<0.001*
-6	30	off	CoE	6	30	off	CoE	0.026	0.009	0.044	<0.001*
-6	30	off	CoE	6	30	on	CoE	0.026	0.007	0.044	<0.001*
-6	30	on	CoE	0	0	off	BL2	0.024	0.005	0.043	0.002*
-6	30	off	CoE	0	10	off	CoE	0.021	0.004	0.039	0.004*
0	10	off	CoE	0	0	off	BL2	0.016	-0.002	0.034	0.112
-6	30	on	CoE	-6	10	on	CoE	0.015	-0.003	0.033	0.250
-6	30	on	CoE	0	30	off	CoE	0.015	-0.004	0.033	0.283
-6	30	on	CoE	-6	10	off	CoE	0.014	-0.004	0.033	0.322
-6	30	off	CoE	-6	30	on	CoE	0.014	-0.005	0.033	0.362
-6	30	on	CoE	6	10	on	CoE	0.014	-0.005	0.032	0.370
-6	30	on	CoE	6	10	off	CoE	0.014	-0.005	0.032	0.401
-6	30	on	CoE	6	30	off	CoE	0.012	-0.006	0.031	0.568
6	30	on	CoE	0	0	off	BL2	0.012	-0.007	0.031	0.628
-6	30	on	CoE	6	30	on	CoE	0.012	-0.008	0.031	0.742
6	30	off	CoE	0	0	off	BL2	0.011	-0.007	0.029	0.669
6	10	off	CoE	0	0	off	BL2	0.010	-0.008	0.028	0.782
6	10	on	CoE	0	0	off	BL2	0.010	-0.008	0.028	0.792
-6	10	off	CoE	0	0	off	BL2	0.010	-0.008	0.027	0.839
0	30	off	CoE	0	0	off	BL2	0.009	-0.009	0.027	0.877

Table S2. (cont. from last page)

Level 1 (L1)				Level 2 (L2)							
Δf_m	A_m	Met.	Exp. Type	Δf_m	A_m	Met.	Exp. Type	L1-L2	Lower CL	Upper CL	*p*
-6	10	on	CoE	0	0	off	BL2	0.009	-0.009	0.027	0.891
0	10	off	CoE	-6	10	on	CoE	0.007	-0.010	0.025	0.960
-6	30	on	CoE	0	10	off	CoE	0.007	-0.011	0.026	0.978
0	10	off	CoE	0	30	off	CoE	0.007	-0.010	0.025	0.972
0	10	off	CoE	-6	10	off	CoE	0.007	-0.011	0.024	0.981
0	10	off	CoE	6	10	on	CoE	0.006	-0.011	0.024	0.989
0	10	off	CoE	6	10	off	CoE	0.006	-0.011	0.024	0.992
0	10	off	CoE	6	30	off	CoE	0.005	-0.013	0.023	0.999
0	10	off	CoE	6	30	on	CoE	0.004	-0.015	0.023	1.000
6	30	on	CoE	-6	10	on	CoE	0.003	-0.015	0.022	1.000
6	30	on	CoE	0	30	off	CoE	0.003	-0.016	0.022	1.000
6	30	on	CoE	-6	10	off	CoE	0.003	-0.016	0.021	1.000
6	30	off	CoE	-6	10	on	CoE	0.002	-0.015	0.020	1.000
6	30	on	CoE	6	10	on	CoE	0.002	-0.016	0.021	1.000
6	30	off	CoE	0	30	off	CoE	0.002	-0.015	0.020	1.000
6	30	on	CoE	6	10	off	CoE	0.002	-0.017	0.021	1.000
6	30	off	CoE	-6	10	off	CoE	0.002	-0.016	0.019	1.000
6	30	off	CoE	6	10	on	CoE	0.001	-0.016	0.019	1.000
6	10	off	CoE	-6	10	on	CoE	0.001	-0.016	0.018	1.000
6	30	off	CoE	6	10	off	CoE	0.001	-0.017	0.019	1.000
6	10	on	CoE	-6	10	on	CoE	0.001	-0.016	0.018	1.000
6	10	off	CoE	0	30	off	CoE	0.001	-0.016	0.018	1.000
6	30	on	CoE	6	30	off	CoE	0.001	-0.018	0.020	1.000
6	10	on	CoE	0	30	off	CoE	0.001	-0.016	0.018	1.000
-6	10	off	CoE	-6	10	on	CoE	0.001	-0.016	0.018	1.000
6	10	off	CoE	-6	10	off	CoE	0.001	-0.017	0.018	1.000
6	10	on	CoE	-6	10	off	CoE	0.000	-0.017	0.018	1.000
-6	10	off	CoE	0	30	off	CoE	0.000	-0.017	0.018	1.000
0	30	off	CoE	-6	10	on	CoE	0.000	-0.017	0.017	1.000
6	10	off	CoE	6	10	on	CoE	0.000	-0.017	0.017	1.000

This table summarizes the results of Tukey's HSD test for data shown in Figure 5a (main text). Each row indicates significance of the difference between least squares means associated with trial conditions in Level 1 (L1) and Level 2 (L2). There are three levels of motor frequency ($\Delta f_m = 0, \pm 6\%$), two levels of oscillation amplitude ($A_m = 10, 30\%$ body weight), two experimental phases where the metronome is either inactive (i.e. Met. = off; $0 < \mathrm{Time} < 300$ s) or active (i.e. Met. = on; $300 < \mathrm{Time} < 600$ s), and three experiment types: Baseline 1 (BL1: the subject walks freely on the treadmill with the harness off), Baseline 2 (BL2: the subject walks freely on the treadmill with the harness on), and a typical experiment

trial (CoE, where the subject interacts with the system at constant frequency and amplitude). All confidence limits (CL) are set at 95% and p values are marked with an asterisk to indicate significance after controlling for multiple testing.
